# Sequence-Based Antigenic Change Prediction by a Sparse Learning Method Incorporating Co-Evolutionary Information

**DOI:** 10.1371/journal.pone.0106660

**Published:** 2014-09-04

**Authors:** Jialiang Yang, Tong Zhang, Xiu-Feng Wan

**Affiliations:** 1 Department of Basic Sciences, College of Veterinary Medicine, Mississippi State University, Starkville, Mississippi, United States of America; 2 Department of Statistics, Rutgers University, Piscataway, New Jersey, United States of America; CSIR-Institute of Microbial Technology, India

## Abstract

Rapid identification of influenza antigenic variants will be critical in selecting optimal vaccine candidates and thus a key to developing an effective vaccination program. Recent studies suggest that multiple simultaneous mutations at antigenic sites accumulatively enhance antigenic drift of influenza A viruses. However, pre-existing methods on antigenic variant identification are based on analyses from individual sites. Because the impacts of these co-evolved sites on influenza antigenicity may not be additive, it will be critical to quantify the impact of not only those single mutations but also multiple simultaneous mutations or co-evolved sites. Here, we developed and applied a computational method, AntigenCO, to identify and quantify both single and co-evolutionary sites driving the historical antigenic drifts. AntigenCO achieved an accuracy of up to 90.05% for antigenic variant prediction, significantly outperforming methods based on single sites. AntigenCO can be useful in antigenic variant identification in influenza surveillance.

## Introduction

Influenza A virus causes both seasonal and pandemic outbreaks, presenting a continuous challenge to public health. Influenza A virus is an RNA virus in the family *Orthomyxoviridae*, and its genome is composed of eight negative stranded RNA segments. Two processes, namely antigenic drift and antigenic shift, drive the antigenic changes of influenza A virus. Antigenic drift is mainly caused by mutations in influenza surface glycoproteins hemagglutinin (HA) and neuraminidase (NA), which are primary targets for host immune systems. Antigenic shift occurs when an influenza strain with antigenically distinct HA and/or NA genes appears, usually resulting from genetic reassortment. Genetic reassortment is the exchange of one or more discrete RNA segments into multipartite viruses when two or more viruses infect the same cell. Antigenic drift events occur more frequently than antigenic shift events.

Vaccine is the primary option in counteracting influenza outbreaks. Antigenic matches between circulating strains and vaccine seed strain is the key to an effective vaccination program. Recently, we developed AntigenBridges, a sequence-dependent influenza antigenicity quantification method [1]. This method identified antigenicity-associated sites using sparse learning and developed a quantification score using single mutations. This same method was shown to be effective in inferring influenza antigenicity up to an accuracy of 83.78%. Compared to other laboratory-based methods such as hemaglutination inhibition (HI) and microneutralization (MN), this sequence-dependent method allowed us to perform large-scale antigenic characterization in influenza surveillance. More importantly, it can facilitate antigenic characterization for those viruses requiring a high biosafety facility, such as H5 and H7 influenza A virus, which generally require BioSafety Level 3 (BSL-3) facility.

During influenza evolution, multiple sites can co-evolve. A recent study on HA1 proteins of H3N2 influenza A viruses from 1968 to 2005 showed that 88 of the 95 substitutions occurred in groups, and two or more of these residues can mutate simultaneously [2]. These multiple simultaneous mutations at antigenic sites cumulatively enhance antigenic drift [2]. Other studies also identified 308 putative pairs of co-evolved amino acid positions [3]. These studies have suggested that the residues under correlated evolution (co-evolution) are more likely to be physically close in the three-dimensional structure of the protein [4]. Because the impacts of these co-evolved sites are not necessarily additive, the single site-based method described in the previous study would need to be optimized. In this study, we developed AntigenCO, a sparse learning method by incorporating evolutionary information. Our results showed that AntigenCO outperformed AntigenBridges, and can reach up to 90.05% predictive accuracy.

## Results

AntigenCO is a new sparse learning algorithm to quantify antigenic distances among influenza A viruses using influenza HA1 protein sequence information. AntigenCO identifies top features determining antigenic profiles embedded in serological data. This feature can be either a single residue, co-evolved residues, or a residue coupled within a certain physical distances in three-dimensional structures of HA protein. We summarized in Table S1 various features and feature parameters. Two validation schemes, namely sequential validation and 5-fold cross-validation process were used in tuning the model parameters, and the top selected features were used for constructing a model for sequence-based antigenic prediction. This framework was applied into H3N2 influenza dataset containing 512 viruses and 133 serums collected between 1968 and 2007 together with the corresponding HA1 protein, and the obtained prediction model was tested on H3N2 influenza viruses from 2002 to 2013. As influenza A viruses have been evolved into antigenic clusters [5], we applied AntigenCO to infer mutations leading to the antigenic drift events among these clusters.

### Single and co-evolved sites driving antigenic changes in H3N2 influenza A viruses

**Figure 1:**
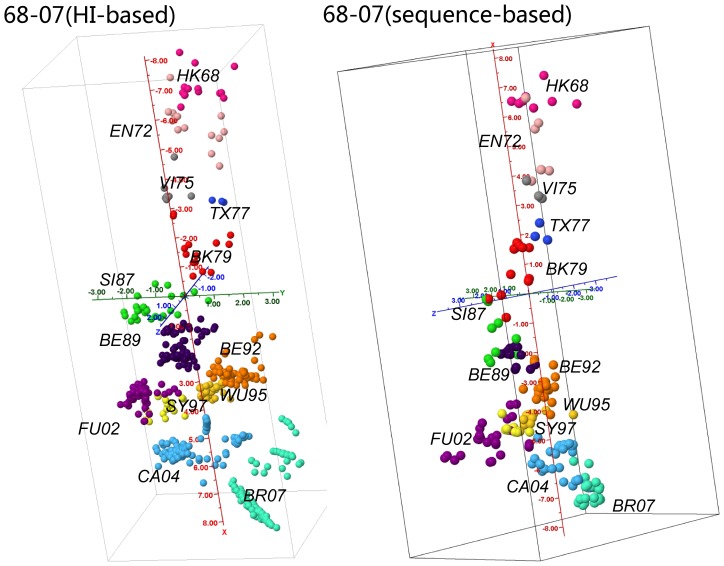
HI-based and sequence-based cartographies on H3N2 68-07 data. Each ball denotes a single influenza virus and each individual color denotes a specific antigenic cluster.

AntigenCO identified 65 antigenicity associated features using H3N2 data from 1968 to 2007 (Table 1) [1]. These features include 13 single sites and 52 pairs of co-evolutionary sites. These features cover 38 residues, including 25, 50(antibody binding site C), 54(C), 57(E), 62(E), 82(E), 83(E), 94(E), 121(D), 126(A), 131(A), 133(A), 137(A), 142(A), 144(A), 145(A), 155(B), 156(B), 157(B), 158(B), 159(B), 160(B), 172(D), 173(D), 189(B), 192(B), 193(B), 196(B), 219(D), 222, 225, 226(D), 244(D), 262(E), 276(C), 278(C), 278(C) and 299(C). Based on these features, the sequence-based antigenic cartography were highly correlated with that from the HI data [1] (Figure 1), with a Pearson correlation coefficient (CC) of 0.94. All the reported antigenic clusters, HK68, EN72, VI75, TX77, BK79, SI87, BE89, BE92, WU95, SY97, FU02, CA04, and BR07 [1], [5], were correctly inferred from sequence-based antigenic cartography.

**Table 1:** Top 65 predominant antigenicity associated sites for H3N2 influenza A viruses.

Site		Weight	Site		Weight	Site		Weight	Site		Weight
225		0.67	276(C)	278(C)	0.31	82(E)	189(B)	0.23	189(B)	225	−0.15
244(D)		0.61	137(A)	155(B)	0.31	142(A)		0.22	173(D)		0.15
158(B)	278(C)	0.50	126(A)	160(B)	0.31	133(A)	142(A)	−0.21	226(D)	262(E)	−0.14
189(B)		0.49	142(A)	144(A)	0.30	226(D)	276(C)	−0.21	131(A)	189(B)	−0.14
225	262(E)	−0.43	133(A)	137(A)	−0.30	159(B)	262(E)	−0.20	159(B)	193(B)	0.13
50(C)	142(A)	−0.42	50(C)	262(E)	0.30	137(A)	276(C)	0.20	159(B)		0.12
189(B)	196(B)	−0.39	83(E)	196(B)	0.28	133(A)	276(C)	0.20	159(B)	192(B)	0.11
83(E)	126(A)	0.38	159(B)	299(C)	−0.27	83(E)	137(A)	−0.20	157(B)	262(E)	0.09
121(D)	278(C)	0.37	50(C)		0.27	142(A)	262(E)	0.19	133(A)	262(E)	0.09
142(A)	278(C)	−0.37	144(A)	156(B)	−0.27	54(C)	173(D)	−0.19	219(D)		−0.09
159(B)	189(B)	0.35	50(C)	278(C)	−0.27	62(E)	299(C)	0.19	133(A)	172(D)	0.09
50(C)	155(B)	−0.35	193(B)	225	0.26	82(E)	94(E)	0.19	121(D)		−0.08
155(B)	225	−0.34	196(B)	278(C)	0.26	189(B)	193(B)	−0.18	57(E)	262(E)	−0.06
193(B)		0.33	126(A)		−0.25	189(B)	278(C)	−0.18	145(A)	189(B)	0.02
222	225	−0.33	25	156(B)	0.25	172(D)		−0.17	159(B)	226(D)	0.02
94(E)	159(B)	0.33	159(B)	225	0.24	226(D)		0.16	159(B)	278(C)	0.01
25	133(A)	0.32									

Weight denotes the importance of the single and co-evolutionary sites in shaping the antigenic evolution. As suggested by the parameter tuning process (Table S2), the sites are generated by feature type “Sinco+EvolT4” and Lasso parameter 2^4^.

**Figure 2:**
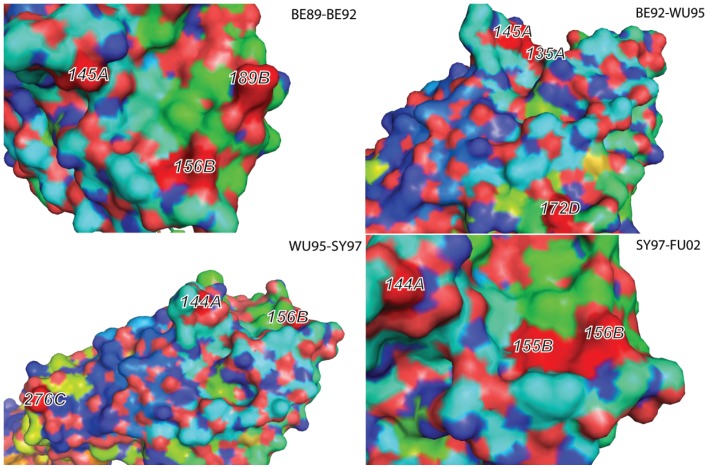
The locations of co-evolutionary sites driving the antigenic drift of four events: “BE89-BE92,” “BE92-WU95,” “WU95-SY97,” and “SY97-FU02.” The H3N2 structure pdb (2VIU) are used as the backbone and the antigenic domains A, B, C, D and E are also marked after the position numbers.

**Figure 3:**
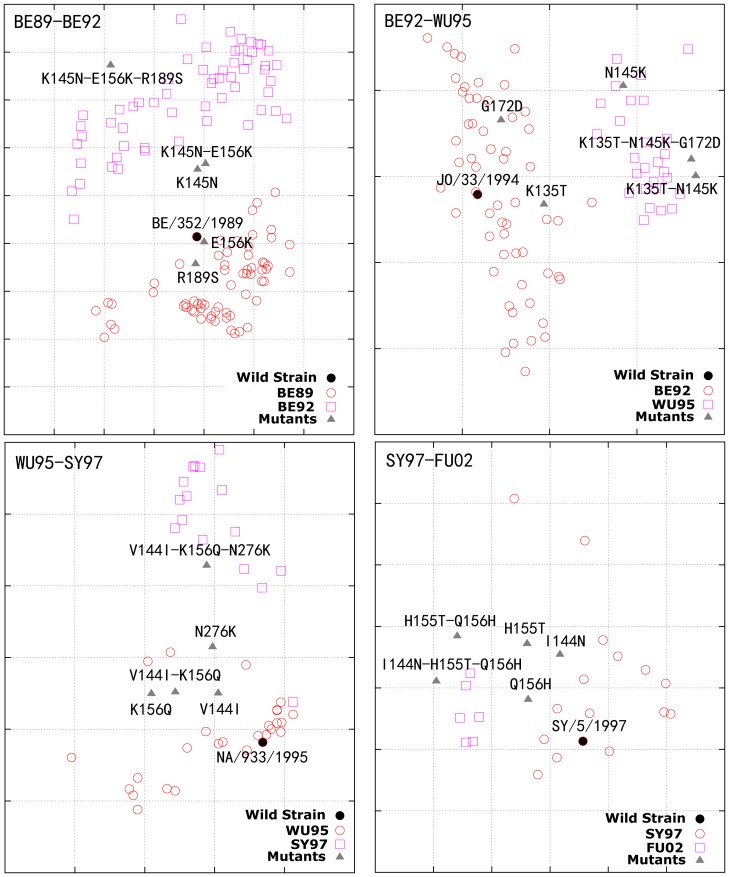
Four simulation cartographies of antigenic drifts and mutants of positions driving the drifts. The four antigenic drift events are: “BE89-BE92,” “BE92-WU95,” “WU95-SY97” and “SY97-FU02”. The mutants listed in Table 2 from four wild strains “BE/352/1989,””JO/33/1994,” “NA/933/1995,” and “SY/5/1997” are also marked in the cartographies.

A set of 12 single or co-mutations were predicted to be responsible for antigenic drifts among these antigenic clusters (Table 2, Figure 2, S1, and S2). Single mutations K156E and S193F were responsible for antigenic drifts TX77->BK79 and CA04->BR07, respectively. The other 10 antigenic drift events were instead driven by co-evolutionary mutations, which can be located at the same antibody binding site or across different antibody binding sites. Antigenic cartography demonstrated those mutations drove antigenic drift among these clusters (Figure 3 and Figure S3).

**Table 2:** Single and co-evolutionary sites driving the 12 antigenic drift events between successive clusters from HK68, EN72, VI75, TX77, BK79, SI87, BE89, BE92, WU95, SY97, FU02, CA04 and BR07.

Drift	Sites	Domain
HK68-EN72	(G144D, N188D)	(A, B)
EN72-VI75	(S145N, S193D, R201K)	(A, B, D)
VI75-TX77	(D53K, E82K)	(C, E)
TX77-BK79	K156E	B
BK79-SI87	(Y155H, V163A, K189R)	(B, B, B)
SI87-BE89	(G135E, N145K, N193S)	(A, A, B)
BE89-BE92	(K145N, E156K, R189S)	(A, B, B)
BE92-WU95	(K135T, N145K, G172D)	(A, A, D)
WU95-SY97	(V144I, K156Q, N276K)	(A, B, C)
SY97-FU02	(I144N, H155T, Q156H)	(A, B, B)
FU02-CA04	(K145N, Y159F, S189N)	(A, B, B)
CA04-BR07	S193F	B

As suggested by parameter tuning process (Table S3 and S4), the sites are generated by feature type “sinco+EvolT8” and Lasso parameter 1; the top numbers are selected by prediction RMSE curve. For simplicity, all top numbers are set to be 10, except for drift EN72-VI75 and BK79-SI87, which is set to be 15, CA04-BR07, which is 3 and SY97-FU02, which is 20.

### Co-evolutionary and structural information increases the accuracy in antigenic distance measurement

**Figure 4:**
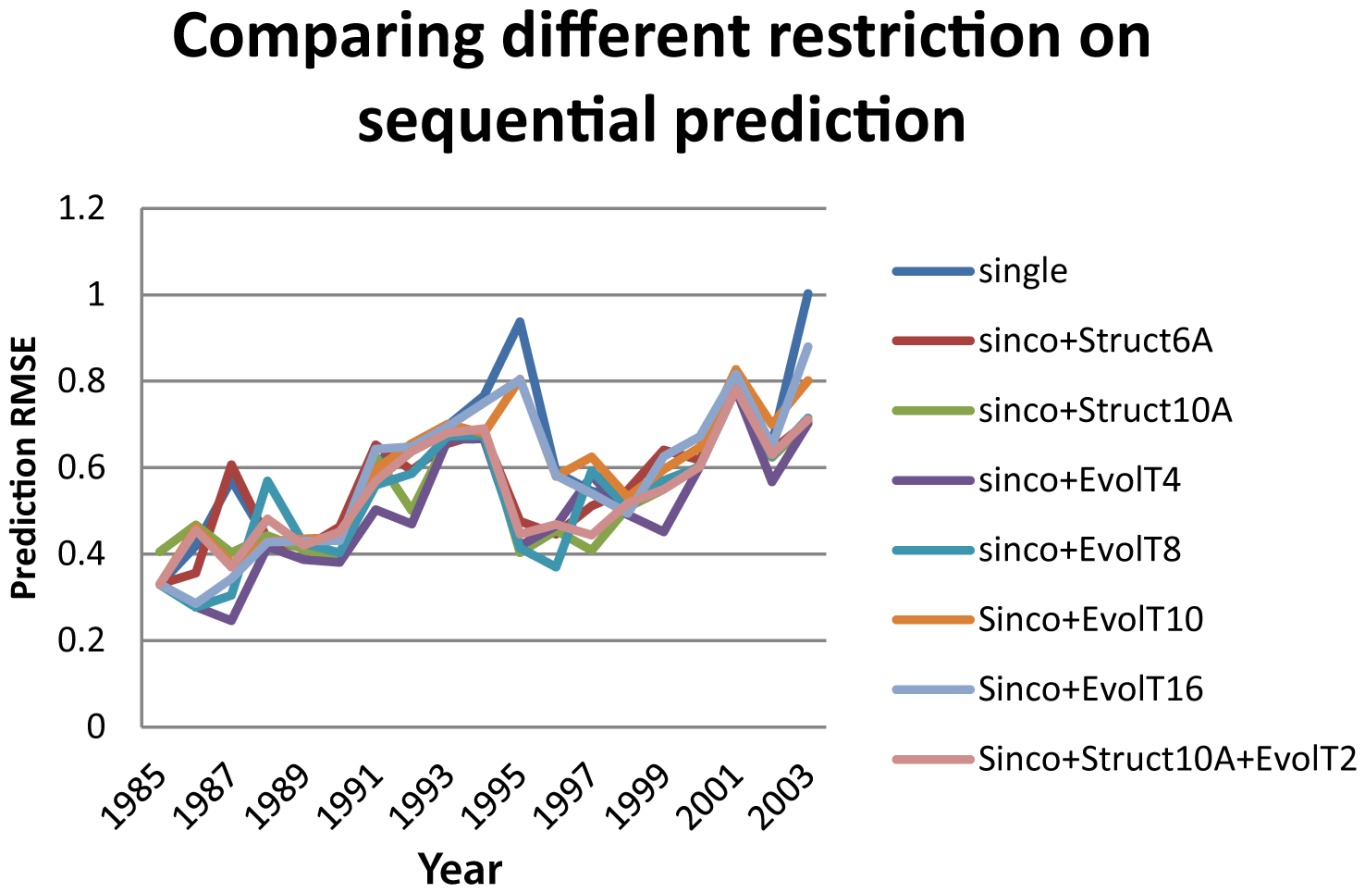
The prediction RMSE curves comparing eight feature types. A sequential prediction was applied for viruses spanning from 1985 to 2003. The 8 feature types are “single”, “sinco+Struct6A”, “sinco+Struct10A”, “sinco+EvolT4”, “sinco+EvolT8”, “sinco+EvolT10”, “Sinco+EvolT16”, and “sinco+Struct10A+EvolT2”.

**Figure 5:**
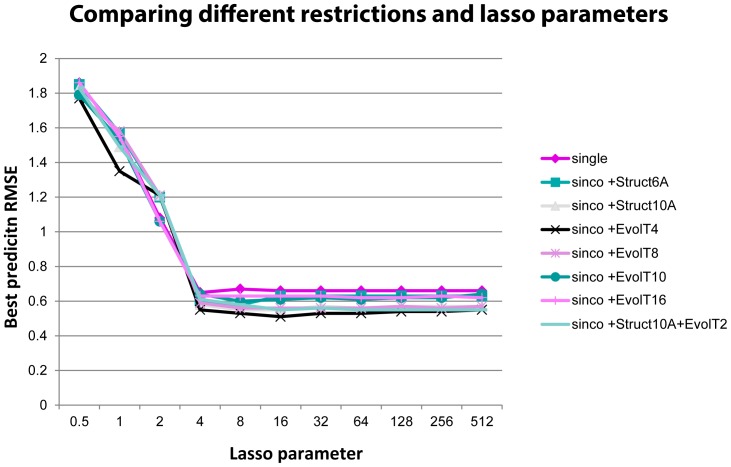
Comparing eight feature types and 11 Lasso parameters.

We compared the prediction accuracies of the separate methods using single sites, co-evolutionary, and co-neighboring sites. Our results showed that both the co-evolutionary information and the co-neighboring information can improve antigenic distance measurement accuracies of sparse learning method, and the co-evolutionary information seemed to be more effective (Figure 4, Table S2). The method combining both co-evolutionary and structural information was also tested, but the prediction accuracy remained similar to those using co-evolutionary information alone. The method “sinco+EvolT4” outperformed all other methods in most years. Comparing to “single” sites, the prediction RMSE of “sinco+EvolT4” decreased by 21.54% on average and decreased up to 60% in some years: 1987, 1995, and 2003. A comparison of different methods and Lasso parameters based on average prediction RMSE from 1985 to 2003 was plotted in Figure 4, Figure 5, and Table S2. The method “sinco+EvolT4” with Lasso parameter 16 is used in future prediction studies.

### The features with co-evolutionary information were more effective than those with single sites in antigenic variant identification

**Figure 6:**
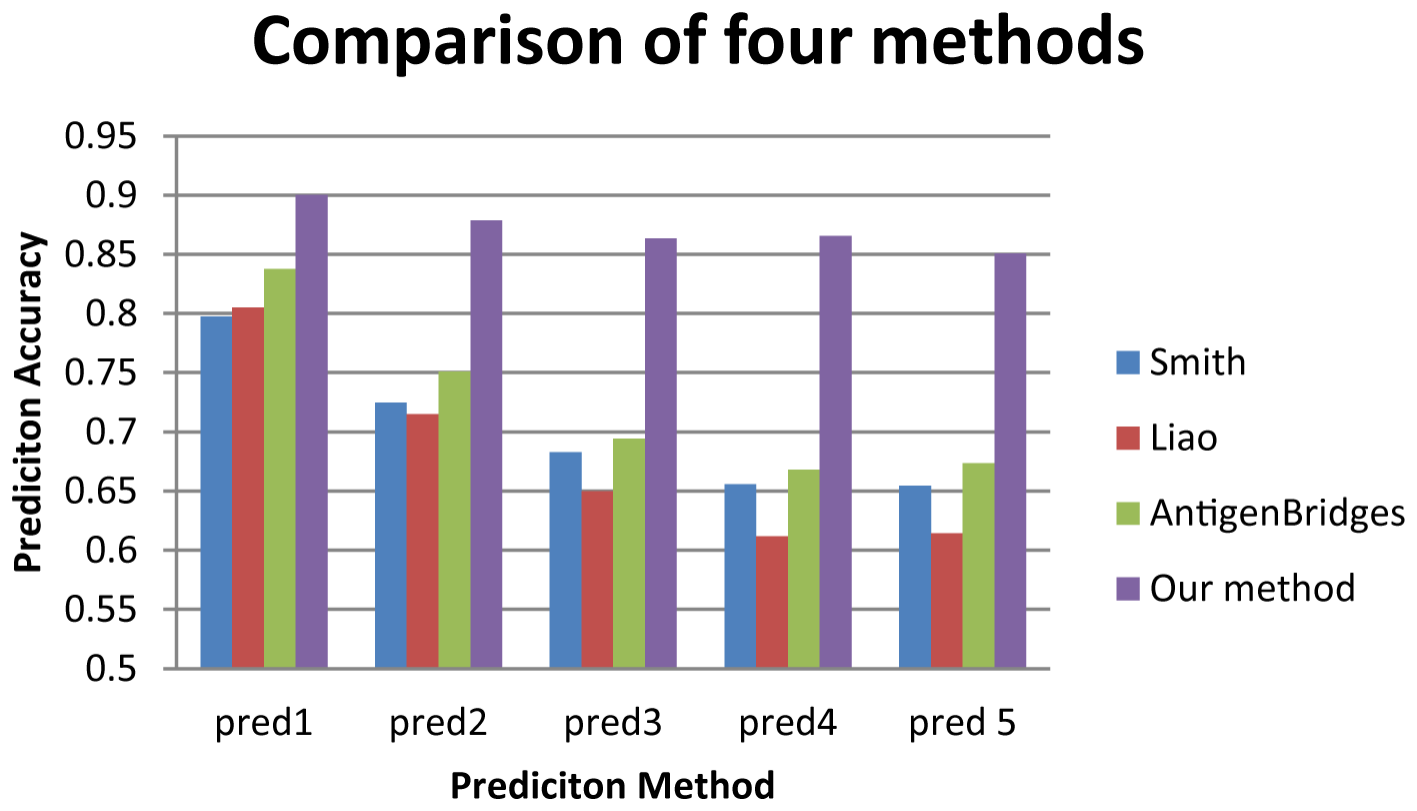
Comparing four methods in predicting antigenic variants. The four methods are Smith et al. [12], Liao et al. [17], AntigenBridges [4] and the sparse learning framework combing co-evolutionary information in this study.

We compared prediction accuracy of the sparse learning framework in this study with three reported feature sets, including 44-single sites [5], 25-single sites [6], and 39-single sites [1]. Our results clearly showed that the feature set integrating co-evolutionary information outperformed the other three feature sets (Table 3 and Figure 6). The improvements were up to 41.1%, 32.4%, 28.7% compared with the method using 44-single sites [5], 25-single sites [6], and 39-single sites [1], respectively.

**Table 3:** Comparing four methods in predicting antigenic variants.

Method	Pred1	Pred2	Pred3	Pred4	Pred5
Smith(weight by AntigenBridges)	0.7975	0.7248	0.6831	0.6560	0.6546
Liao(weight by AntigenBridges)	0.8051	0.7151	0.6501	0.6117	0.6143
AntigenBridges	0.8378	0.7510	0.6942	0.6683	0.6736
AntigenCo	0.9005	0.8787	0.8637	0.8658	0.8666

Five accuracies “Pred1”, “Pred2”, “Pred3”, “Pred4” and “Pred5” were used to show prediction accuracies for 1, 2, 3, 4 and 5 seasons. “Pred1” predicted the pairwise distances of viruses in each pair of consecutive years *k* and *k−*1 for 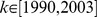 using viruses in [1968, *k*−1] as training data. “Pred2” predicted the distances between viruses in year *k* and *k*−1, and between viruses in year *k*−2 and those in years *k* and *k*−1 using viruses in [1968, *k*−2] as training data. Similar definitions hold for “Pred3”, “Pred4”, and “Pred5”.

### Sequence based antigenicity predication using co-evolutionary sites

**Figure 7:**
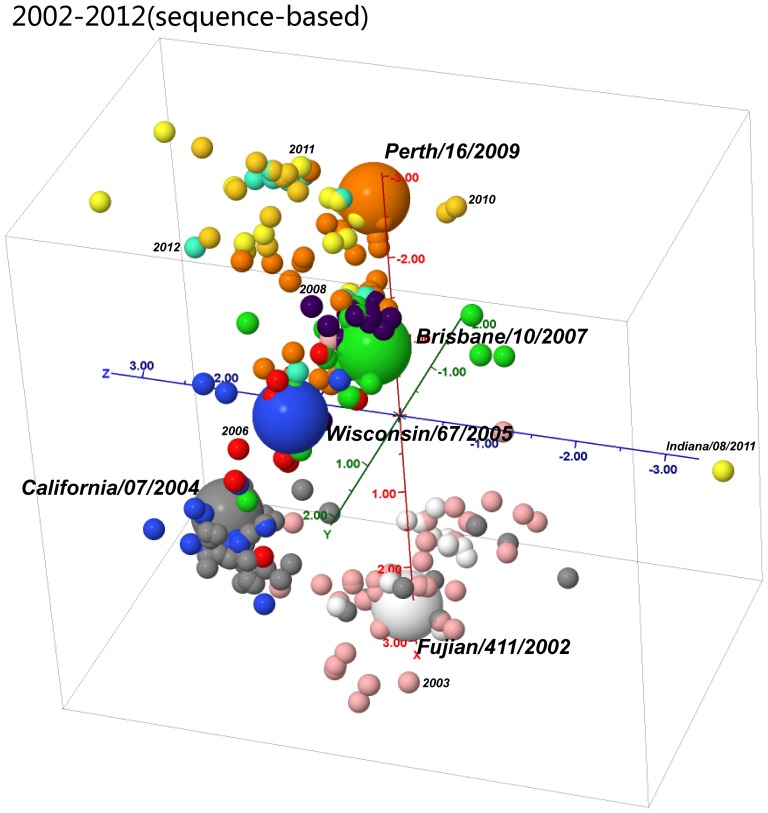
Sequence-based cartographies on 1415 H3N2 influenza viruses from 2002 to 2013 downloadable from NCBI. Each colored ball represents a virus. The different colors mark its collection year. The five vaccine strains “Fujian/411/2002,” “California/07/2004,” “Wisconsin/67/2005,” “Brisbane/10/2007,” and “Perth/16/2007” are shown in big ball. We also mark the year of a representative virus in other years.

A total of 1,415 non-redundant HA1 sequences of H3N2 viruses from 2002 to 2013 were collected. To show the effectiveness of AntigenCO, the antigenic distances among these viruses were quantified based on features derived from the H3N2 data from1968 to 2007 [1]. Figure 7 shows that there are four clear antigenic drift events in years 2003, 2005, 2007, and 2009, which are reported previously as “FU02->CA04,” “CA04->WI05,” “WI05->BR07,” and “BR07->PE09” [7]. In addition, the cartography shows that there is a small antigenic distance between viruses before and after 2011, and the viruses after 2011 had a large extent of antigenic variations.

## Discussion

Antigenic changes in seasonable influenza viruses were recently shown to occur more gradually by our recent study [1] and others [2]. These results suggested that multiple mutations in antibody binding sites can occur but not necessarily simultaneously. Some antigenic drift events were driven by multiple mutations, and the impacts of these mutations on antigenic changes are not necessarily additive. For example, our previous experiment showed that mutation N145K and G172D from virus JO/33 changed antigenic distance by 1.29 and 0.44 unit respectively. The N145K-G172D double mutation resulted in an antigenic distance change of 1.83 units, which are different from the simple sum of the antigenic distances from two corresponding individual mutations [1]. This motivated us to improve our earlier prediction functions of influenza A viruses in this study by incorporating features with the co-evolved residues in addition to those single residues. Our results confirmed that incorporation of coupled residues into the prediction function does improve the predictive function of antigenic distances.

This study identified 65 features derived from 38 individual residues contributing to antigenic changes of H3N2 influenza A viruses. These residues included 13 single sites and 52 pairs of co-evolutionary sites (Table 1). Except for site 244, 12 out of these 13 single sites were identical to those identified from our previous study [1]. The learning results demonstrated that the impacts of these residues on antigenic drift are not additive, confirming our hypothesis. Nevertheless, the performance of the new predictive function incorporated evolutionary information has been significantly improved.

Our previous study suggested multiple mutations leading to a single antigenic drift event can occur at not only the same antibody binding site but also more than one antibody binding site [1]. In this study, we evaluated the co-evolved residues within the same binding site and those across multiple antibody binding sites. Our results further confirmed our previous study and are also consistent with those reported recently [8]. From 1968 to 2007, only two of those 12 historical antigenic drift events, TX77-BK79 and CA04-BR07, were caused by a single mutation whereas the other 12 by two or three residues within or across at least two antibody binding sites (Table 2).

In summary, this study developed a predictive function, AntigenCO, in quantifying antigenic changes using antigenicity-associated sites derived from HI results by sparse learning. The impacts of individual residues on antigenic changes were shown to be non-additive. AntigenCO incorporates such information and achieved an accuracy of up to 90.05% for antigenic variant prediction.

## Materials and Methods

### Benchmark data set

The H3N2 HI table, containing 512 viruses and 133 serums collected between 1968 and 2007 together with the corresponding HA1 protein sequences [1], was used as the benchmark data. The 512 H3N2 viruses were grouped into 13 clusters: “HK68,” “EN72,” “VI75,” “TX77,” “BK79,” “SI87,” “BE89,” “BE92,” “WU95,” “SY97,” “FU02,” “CA04”, and “BR07,” and their HA protein sequences were downloaded from NCBI. To study the antigenic drift between two clusters, for example “BE92-WU95,” we retrieved the HI and sequence data from the viruses in the two clusters “BE92” and “WU95”. The viruses in other clusters did not have a direct contribution to the antigenic drift event. For convenience, the 13 antigenic drift sub-data sets were named the same as the antigenic drift. We also downloaded 1,415 HA protein sequences from 2002 to 2013 to test the prediction ability of the sparse learning framework with co-evolutionary information.

### AntigenCO

AntigenCO is a sparse learning algorithm to quantify antigenic distance among influenza A viruses using influenza HA1 protein sequence. We implemented AntigenCO in Matlab and all source codes and data used for this study are available at http://sysbio.cvm.msstate.edu/AntigenCO.

#### Sparse learning algorithms

Specifically, the pairwise antigenic distances among viruses are measured by antigenic cartography [9] based on a serological dataset, e.g. HI data. Let the number of viruses be N, then the distances could be formulated as a vector *Y* of length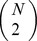, in which each entry calculates the antigenic distance between a pair of viruses. And similarly, we used a matrix 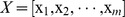 to model the genetic profile, where m is the number of single and correlated co-sites, and *x_i_* is a vector of length 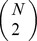 representing the pairwise genetic change at single or correlated co-site *i*. To identify antigenicity-associated sites, we mapped the antigenic distances to genetic profile and selected sites whose mutations shape antigenic vector *Y*. This is a typical feature selection problem in machine learning. Methods like Lasso [10], [11] and Ridge regression [11] are effective for selecting a small to moderate number of antigenicity-associated single and correlated co-sites.

The Lasso [10] formulates the problem as
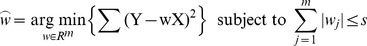
(1)where *s* is a threshold parameter that can be tuned to optimize accuracy and 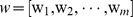 denotes the weights of each single and co-evolved sites. Similarly, the Ridge regression formulation of the problem is



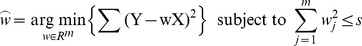
(2)Lasso is solved by the Matlab code from Sköglund [12] and Ridge regression by the Matlab built-in function “ridge.m.”

For our H3N2 data, Lasso performs slightly better in prediction root-mean-square error (RMSE) than Ridge regression in two randomly selected antigenic drift data and three sequential data (Table S5). Thus, we adopted Lasso to perform the analysis throughout this study.

#### Construction of feature vector and scoring schemes

To generalize the single feature in Sun et. al [1], we introduced co-features modeling co-mutations at two sites and then combined single and co-features to model the contribution of both types of sites. There are three types of feature selection methods “single,” “co”, and “sinco”. The feature type “single” takes each single site as a feature and does not consider their correlations; the feature “co” considers each pair of single sites as a feature and only considers their co-mutation information; the feature “sinco” combines the “single” and “co” features and models both the contribution of single amino acid sites and their correlations to antigenic evolution. The residues on the surface of HA protein play central roles in shaping antigenic evolution and co-evolutionary residues [5] and structural co-neighboring residues work together to define protein functions [4]. Thus, here, the feature vectors only include the residues located at the HA surface, which either co-evolve or are physically close in the protein structure.

#### Feature encoding functions

We adopted two-feature encoding schemes in this study, binary and PIMA (Figure S4) scoring schemes, as described in our previous study [1]. A simple comparison in Table S5 shows that the performance of PIMA slightly outperforms that of binary scheme, and thus PIMA will be used in this study.

#### Single feature

The construction of the single feature is the same as described by Sun et. al [1]. Specifically, let *S* be the HA1 protein sequence alignment of *N* viruses. Then, the single feature represents site *i* is an 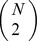 vector 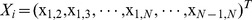, where *x_a,b_* is the binary or PIMA score of a pair of amino acids *S_a,i_* and *S_b,i_* for 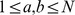 and *S_a,i_* denotes the amino acid of virus *a* at position *i*.

#### Co- and sinco feature

Let *i* and *j* be two sites and *X_i_* and *X_j_* be their representing single features. We construct the co-feature of site *i* and *j* by the inner product of vector *X_i_* and *X_j_*. For example, if the vectors of the “single” features are 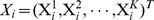
and 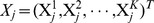, then the corresponding “co-feature” is 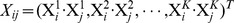. This “co-feature” models the co-occurrence of mutations in site *i* and *j*. The “sinco” feature are those integrating both “single” and “co-” features.

#### Identifying surface sites and structural co-neighboring sites

Residues predicted to be on the surface of the HA homotrimer were determined as described previously [1]. Jmol (www.jmol.org) was used to identify amino acid residues having distances less than a predetermined distance threshold from 1 to 10 angstrom with step 1 from H3N2 HA structure (pdb file 2VIU). By structural co-neighbor restriction, we only allowed the co-features with amino acid pairs having distances less than the threshold. In Figure S5, the curve shows the number of neighboring pairs against the distance threshold from 1 to 10. A pre-analysis showed that threshold around 6 to 10 (data not shown) maximized the prediction accuracy, and we selected two thresholds 6 and 10 for further analysis. The two methods are denoted as “sinco+Struct6A” and “sinco+Struct10A.”

#### Mutual information to identify co-evolutionary sites

It has previously been shown that co-evolution at antigenic sites cumulatively enhances antigenic drift [2]. We adopted simple mutual information methods to identify co-evolutionary amino acid sites:

Let 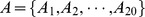 be the amino acid set. Then, the entropy of a single amino acid site *x* is defined to be
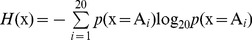
(3)where 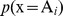 denotes the frequency of amino acid *A_i_* at site *x*.

The joint entropy of two sites *x* and *y* is defined as
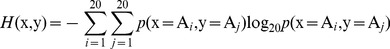
(4)where 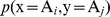 denotes the frequency of amino acid A*_i_* at site *x* and amino acid A*_j_* at site *y* simultaneously.

The mutual information of site *x* and *y* is then defined as
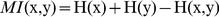
(5)


Martin et. al [13] previously showed that 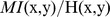 removes the background information and outperforms others, thus we adopted this normalization scheme. After calculating the mutual information for all pairs, we calculated its 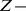
score of the mutual information of a pair of sits *x* and *y* as 
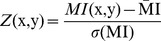
, where 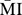 denotes the mean value of the mutual information of all pairs and 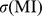 denotes the standard deviation of mutual information. A threshold value was set to *Z* to determine co-evolutionary pairs. Figure S6 shows the correlation between the number of co-evolutionary pairs inferred and the thresholds of Z-value for the benchmark 512 sequence from 1968 to 2007. A simple cross-validation analysis showed that the best threshold was from 4 to 16 based on prediction RMSE (data not shown). Thus, we selected thresholds 4, 8, 10, and 16 for further analysis and the methods are denoted as “sinco+EvolT4,” “sinco+EvolT8,” “sinco+EvolT10,” and “sinco+EvolT16” respectively.

#### Combining co-evolutionary and structural information

It is natural to combine co-evolutionary and structural information as they may function together. In the study, we only tested one method combining “sinco+EvolT2” and “sinco+Struct10A,” which is denoted as “sinco+Struct10A+EvolT2.” Figure 5 illustrates how this method is outperformed by “sinco+EvolT4.” We believe that the combination of co-evolutionary and structural information may not increase the prediction accuracy significantly. Thus, we did not perform a deeper analysis.

#### Determining the top number of features

The top number of features of sequential prediction and antigenic drifts are determined by the prediction RMSE curve against the number of features (see Figure S7, S8, and S9). The two figures show that 10–15 features would be enough for antigenic drift data and 30–65 features work well for sequential prediction.

#### Sequence-based antigenic distance predicting function

Using Lasso, suppose we selected *p* antigenicity-associated single and co-sites and their associated weights. For simplicity, the *p* single and co-sites are re-labeled from 1 to *p*. We quantified the antigenic distance using the function
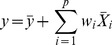
(6)where 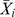 is the normalized features on single or co-site *i*, 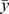 is the mean antigenic distance in the training set, and *w_i_* is the weight assigned to each selected feature *i*. The predicted antigenic distances of the viruses are then plotted into two-dimensional or three-dimensional cartography using a multidimensional scaling method (Figure 1, 3, and 7).

### Selecting top single or co-evolutionary sites for antigenic drifts

In selecting the top single or co-evolutionary sites for antigenic drift events, we first applied our sparse learning framework with co-evolutionary information into the drift data, obtaining the weights for both single and co-sites. Then, we searched complete graphs in the co-evolutionary file, adding the weights up for all the single and double sites. For example, the weight of co-evolutionary site 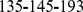 is defined as 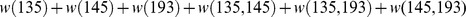



Currently, we have only identified co-evolutionary sites of sizes up to 3. In the end, we ranked all the cliques and selected the one with the highest weight as the single or co-evolutionary sites responsible for the antigenic drift (see Table 2).

### Evaluation methods and parameter tuning

Similar to [1], the root mean square error (RMSE) and Pearson correlation coefficient (CC) were used as measures of prediction and training accuracy for tuning best model parameters, e.g. Lasso and Ridge parameter, feature types, and top number of single or co-sites to choose. Specifically, the Lasso and Ridge parameter was tuned from 2^−10^ to 2^10^ with a multiple of two. In addition, we compared single feature alone, “sinco+Struct*n*A” with *n* from 2 to 10, “sinco+EvolT*m*” with *m* being 0, 2, 4, 8, 10, 16 and 32, and “sinco+Struct10A+EvolT2”. For brevity, we only showed the prediction accuracy of representative feature types and parameters, for example, “sinco+EvolT4” and “2^−1^”.

We applied two types of validation methods, namely 5-fold cross-validation and sequential validation for antigenic drift data and the whole H3N2 data (See Figure S10), respectively. For the 5-folder cross-validation, we randomly selected 20% viruses as a testing set and the remaining 80% viruses as a training set. Then, we examined the true and predicting distances within viruses in the testing set as well as between testing viruses and training viruses. To avoid the influence of randomness, we reran the program 100 times and used the mean RMSE as the criterion. For sequential parameter tuning, five schemes (Pred1, Pred2, Pred3, Pred4, and Pred5) were applied. Pred1 predicted the pairwise distances of viruses in each pair of consecutive years *k* and *k*−1 for 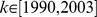 using viruses in [1968, *k*−1] as training data. Pred2 predicted the distances between viruses in year *k* and *k*−1, and between viruses in year *k*−2 and those in years *k* and *k*−1 using viruses in [1968, *k*−2] as training data. Similar definitions hold for Pred3, Pred4, and Pred5.

### Antigenic cartography

The two-dimensional antigenic cartography was constructed by AntigenMap [9] and the three dimensional cartography by AntigenMap3D [14], where the lower reactor was set to 20. The distance-based cartography was constructed using Matlab's built-in nonmetric multidimensional scaling function, “mdscale.m”. The downloaded sequences were aligned using MUSCLE [15].

## Supporting Information

Figure S1 **The mutation pattern of sites responsible for 12 antigenic drifts.** The pattern is marked by year; the amino acids in blue indicate the dominate amino acids in the former antigenic cluster; those in red indicate the dominate amino acids in the later antigenic clusters; and those in yellow are in the middle.
(TIF)

Figure S2 **The position of co-evolutionary amino acid position driving the antigenic 10 drift events on the structure (pdb: 2VIU).**
(TIF)

Figure S3 **Simulation cartographies of single and multiple mutants driving 10 antigenic drifts.** The wild strains are marked in dark solid circle; the mutants driving the antigenic drifts are marked in solid triangles; the viruses in the former antigenic cluster are in red circles; and those in the later cluster are in blue squares.
(TIF)

Figure S4 **PIMA hierarchical scoring function.** The mutation score from an amino acid, e.g. “I” to another amino acid, e.g. “M” is calculated as 6 minus the cardinality of the most recent ancestor of the two amino acids, e.g. “c”. Thus the mutation score between “I” and “M” is 4, i.e. 6 minus 2.
(TIFF)

Figure S5 **Numbers of neighboring pairs with the increase of distances.** The structure file (pdb: 2VIU) is used in the measurement and the distances vary from 0.5 Å to 10 Å with a gap of 0.5 Å.
(TIF)

Figure S6 **Number of co-evolutionary pairs with the increase of Z-score threshold in mutual information analysis.** 512 sequence from 1968 to 2007 are used for the analysis.
(TIF)

Figure S7 **Prediction RMSE curves with the increase of the number of selected features for 12 antigenic drift events.** 5-folder cross validation is used and the RMSE is averaged for 100 bootstrap runs for each antigenic drift event.
(TIF)

Figure S8 **The average prediction RMSE from 1985 to 2003 against the number of sites.** For convenience, the number of sites are shown from 1 to 100 with a gap of 5.
(TIF)

Figure S9 **Training RMSE and CC curve on H3N2 influenza data from 1968 to 2007 against number of sites.** For convenience, the number of sites are shown from 1 to 211 with a gap of 5.
(TIF)

Figure S10 **The prediction RMSE curves comparing different Lasso parameters.** The prediction RMSE curve plots the trend of prediction RMSE from year 1985 to 2003.
(TIF)

Table S1 **Summary of the features used in this study.**
(DOC)

Table S2 **Compare the influence of restriction method and lasso parameter on average prediction RMSE from 1985 to 2003.** Each cell records the average prediction RMSE of the corresponding Lasso parameter, e.g. “2^−1^” and restriction method, e.g. “Single” on sequential prediction data from 1985 to 2003 (see section “Parameter tuning” in “Materials and Methods” for the definition of sequential prediction).
(DOC)

Table S3 **Compare the influence of restriction methods on antigenic drifts.** Each cell records the prediction RMSE of the corresponding restriction method, e.g. “Single” on antigenic drift data, e.g. “HK68-EN72”. To avoid randomness, the RMSE are averaged over 100 runs. In each run, we perform a 5 folder cross validation. For brevity, “6A” indicates co-neighbor restriction with distance 6 angstrom, and “T4” indicates evolutionary restriction with Z-score threshold 4. Similar definition applies for other methods.
(DOC)

Table S4 **Compare the influence of lasso parameter on antigenic drifts.** Each cell records the prediction RMSE of the corresponding lasso parameter, e.g. “2^−1^” on antigenic drift data, e.g. “HK68-EN72”. To avoid randomness, the RMSE are averaged over 100 runs. In each run, we perform a 5 folder cross validation.
(DOC)

Table S5 **Comparison of 2 machine learning methods Lasso and Ridge regression and 2 scoring schemes 0–1 and PIMA.** Each cell lists the smallest average prediction RMSE for all feature types and model parameters on drift data “HK68-EN72” and “BE92-WU95”, and sequential data [1968, 1985], [1968, 1986] and [1968, 1987].
(DOC)
