# Supplementary figures and images for: Sequence-Based Antigenic Change Prediction by a Sparse Learning Method Incorporating Co-Evolutionary Information
Source: PLoS One. 2014 Sep 4;9(9):e106660. doi: 10.1371/journal.pone.0106660 (PMC4154722; doi:10.1371/journal.pone.0106660)

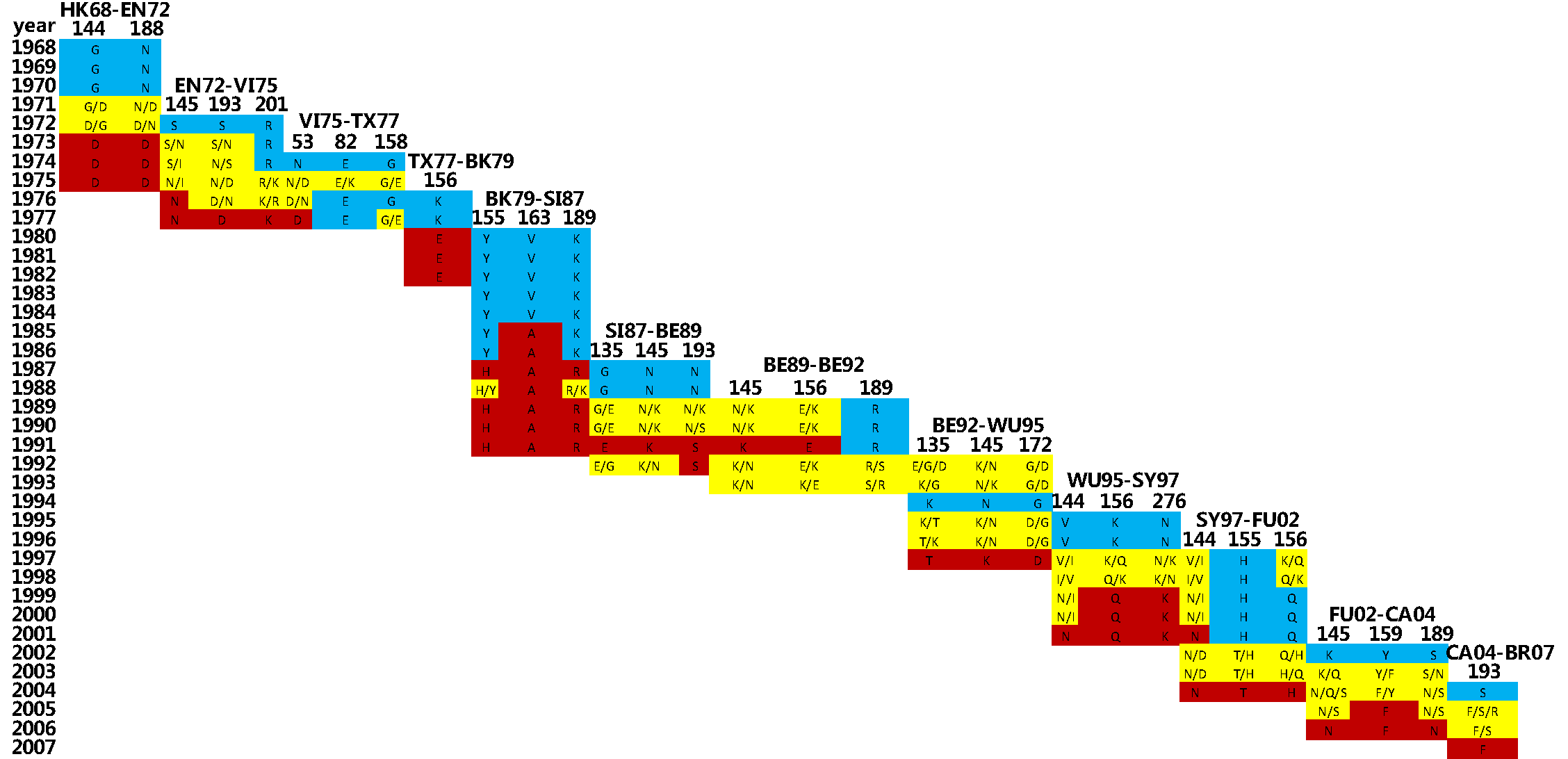

Supplement: Figure S1 — The mutation pattern of sites responsible for 12 antigenic drifts. The pattern is marked by year; the amino acids in blue indicate the dominate amino acids in the former antigenic cluster; those in red indicate the dominate amino acids in the later antigenic clusters; and those in yellow are in the middle. (TIF) [file pone.0106660.s001.tif]

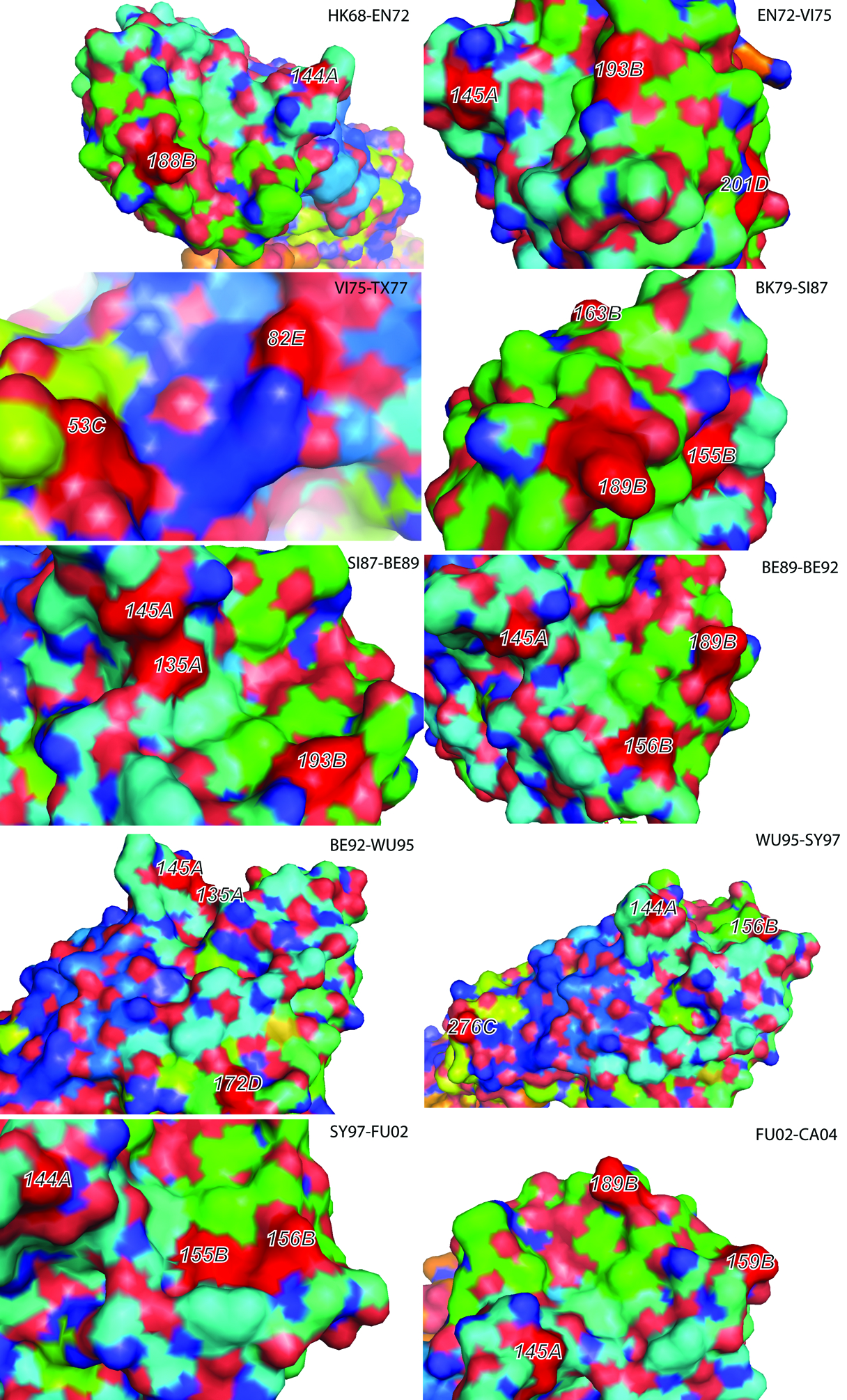

Supplement: Figure S2 — The position of co-evolutionary amino acid position driving the antigenic 10 drift events on the structure (pdb: 2VIU). (TIF) [file pone.0106660.s002.tif]

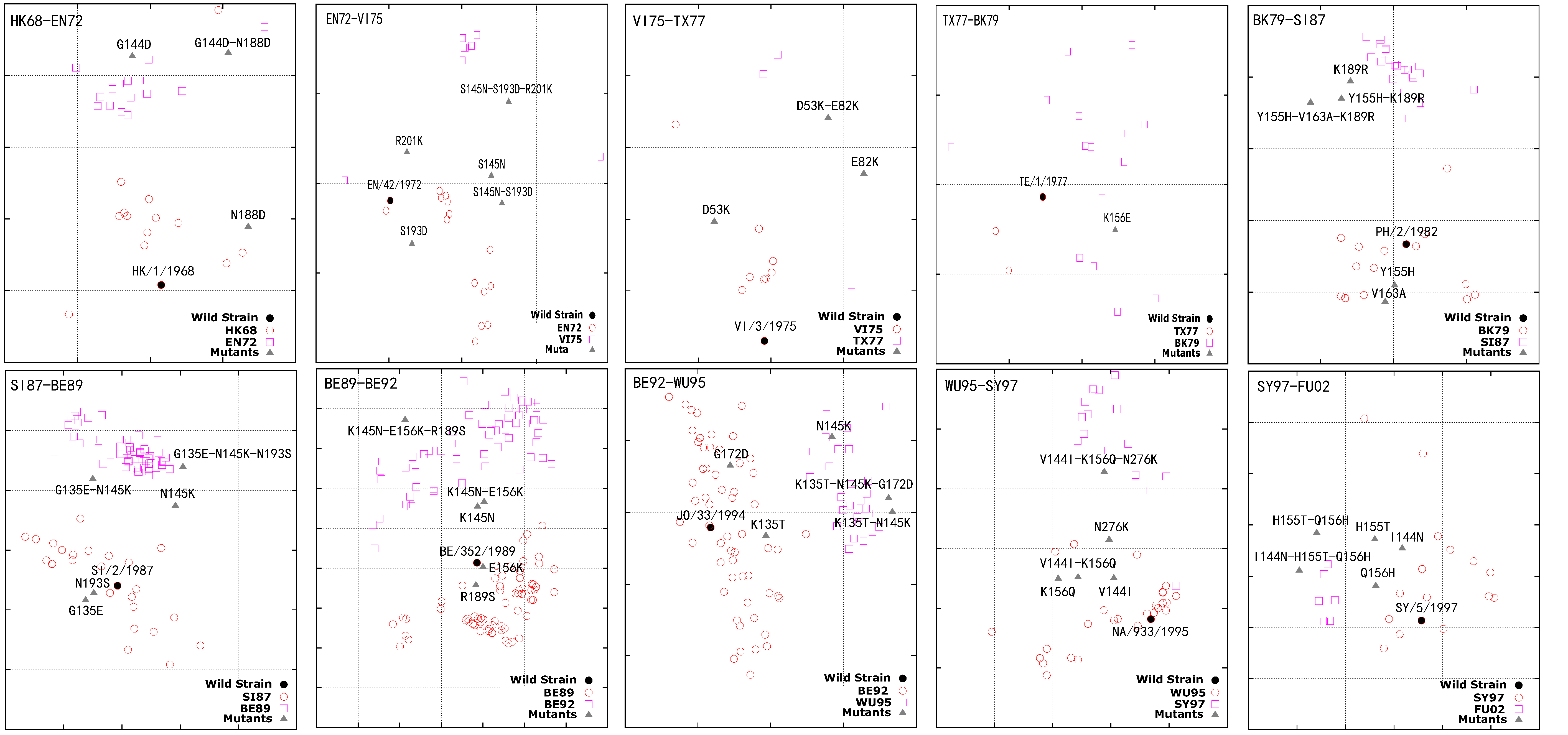

Supplement: Figure S3 — Simulation cartographies of single and multiple mutants driving 10 antigenic drifts. The wild strains are marked in dark solid circle; the mutants driving the antigenic drifts are marked in solid triangles; the viruses in the former antigenic cluster are in red circles; and those in the later cluster are in blue squares. (TIF) [file pone.0106660.s003.tif]

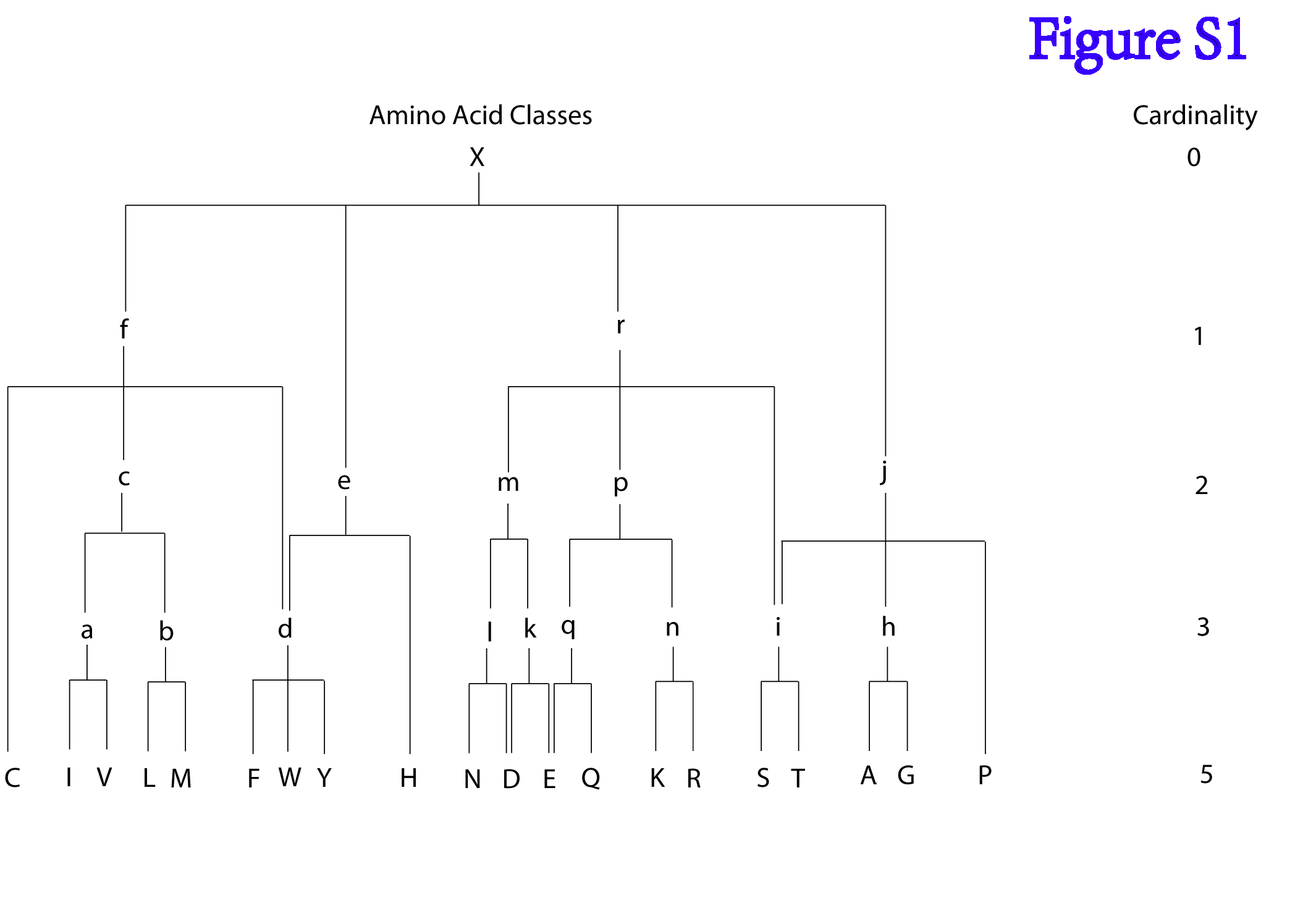

Supplement: Figure S4 — PIMA hierarchical scoring function. The mutation score from an amino acid, e.g. “I” to another amino acid, e.g. “M” is calculated as 6 minus the cardinality of the most recent ancestor of the two amino acids, e.g. “c”. Thus the mutation score between “I” and “M” is 4, i.e. 6 minus 2. (TIFF) [file pone.0106660.s004.tiff]

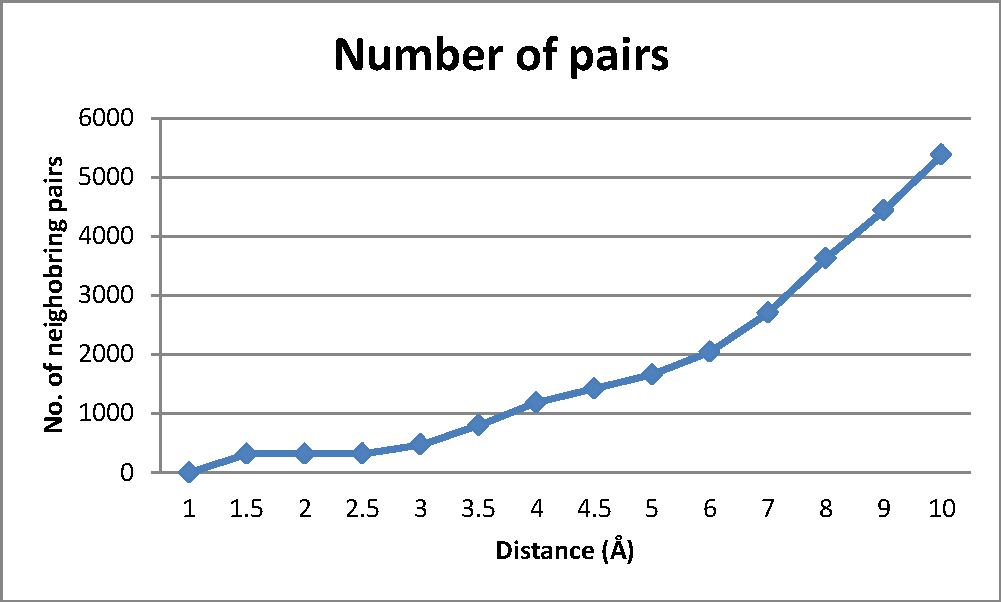

Supplement: Figure S5 — Numbers of neighboring pairs with the increase of distances. The structure file (pdb: 2VIU) is used in the measurement and the distances vary from 0.5 Å to 10 Å with a gap of 0.5 Å. (TIF) [file pone.0106660.s005.tif]

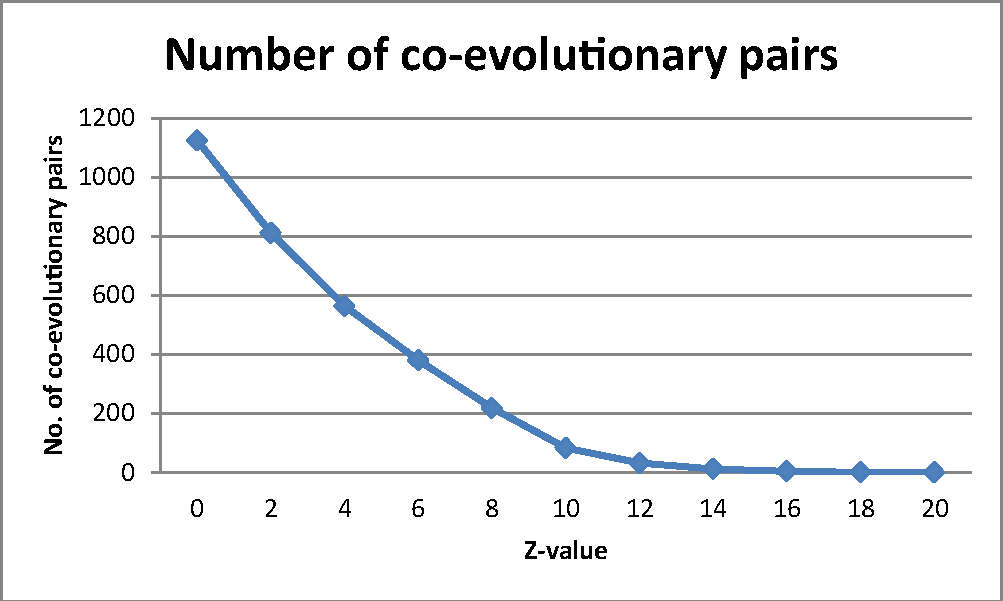

Supplement: Figure S6 — Number of co-evolutionary pairs with the increase of Z-score threshold in mutual information analysis. 512 sequence from 1968 to 2007 are used for the analysis. (TIF) [file pone.0106660.s006.tif]

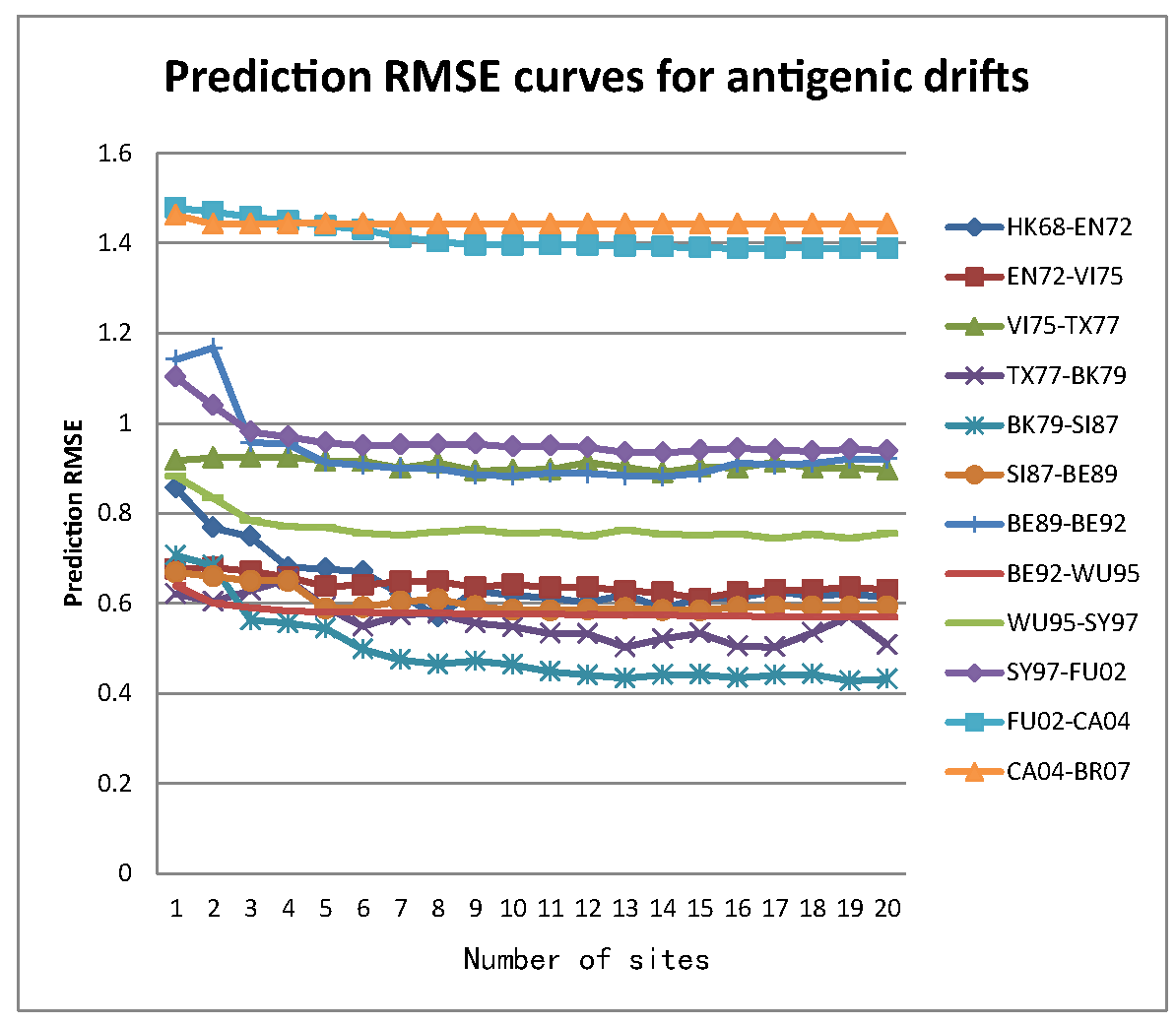

Supplement: Figure S7 — Prediction RMSE curves with the increase of the number of selected features for 12 antigenic drift events. 5-folder cross validation is used and the RMSE is averaged for 100 bootstrap runs for each antigenic drift event. (TIF) [file pone.0106660.s007.tif]

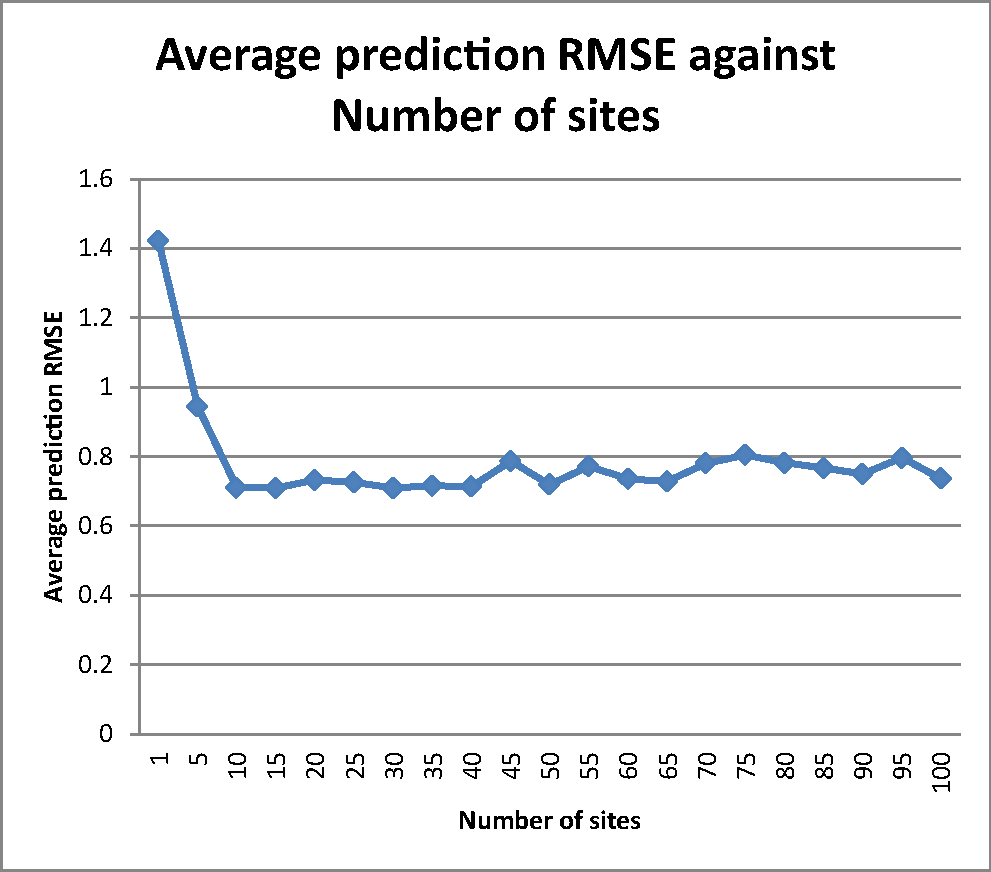

Supplement: Figure S8 — The average prediction RMSE from 1985 to 2003 against the number of sites. For convenience, the number of sites are shown from 1 to 100 with a gap of 5. (TIF) [file pone.0106660.s008.tif]

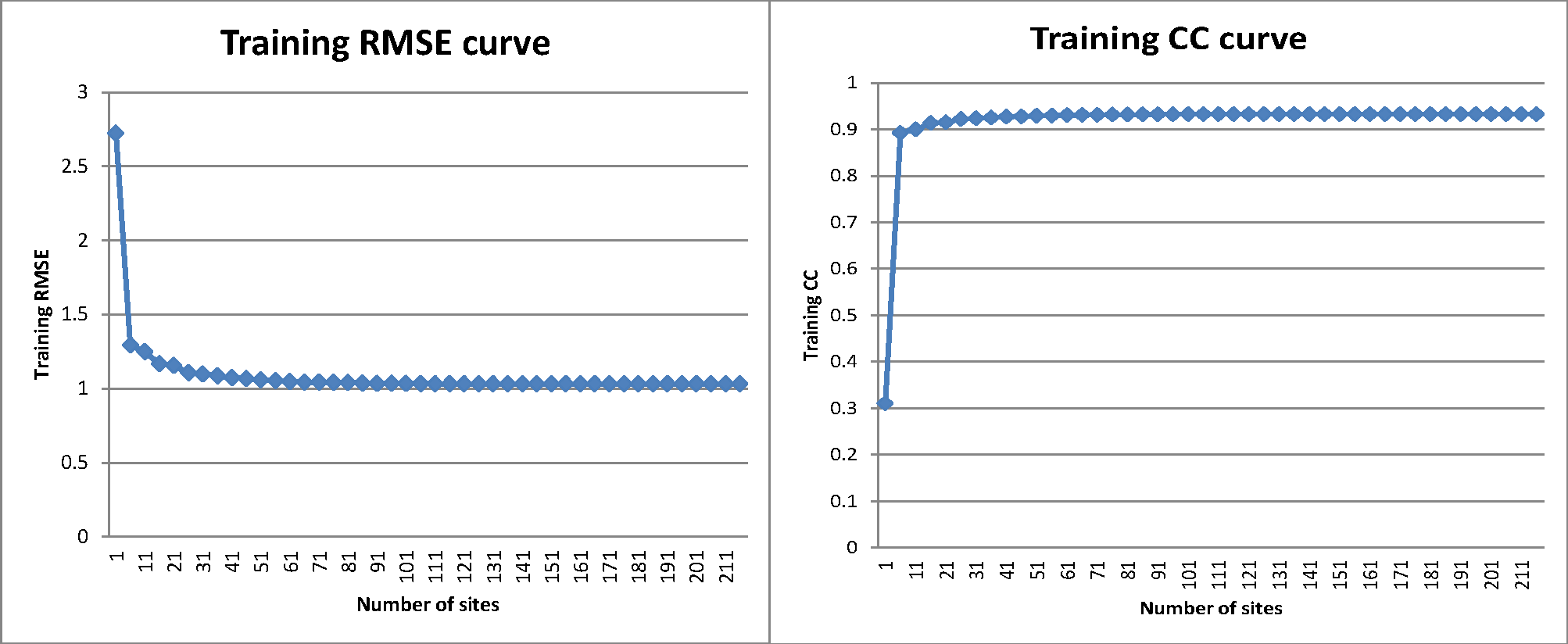

Supplement: Figure S9 — Training RMSE and CC curve on H3N2 influenza data from 1968 to 2007 against number of sites. For convenience, the number of sites are shown from 1 to 211 with a gap of 5. (TIF) [file pone.0106660.s009.tif]

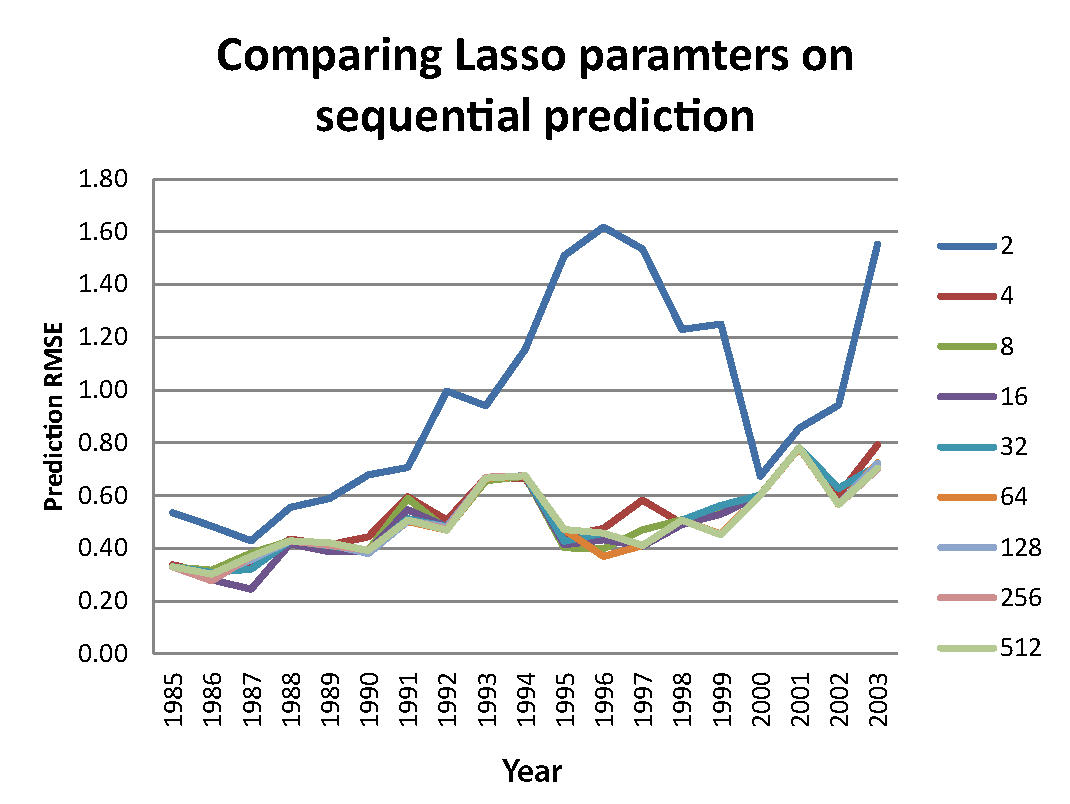

Supplement: Figure S10 — The prediction RMSE curves comparing different Lasso parameters. The prediction RMSE curve plots the trend of prediction RMSE from year 1985 to 2003. (TIF) [file pone.0106660.s010.tif]
