# Supplementary material for: Sequence-Based Antigenic Change Prediction by a Sparse Learning Method Incorporating Co-Evolutionary Information
Source: PLoS One. 2014 Sep 4;9(9):e106660. doi: 10.1371/journal.pone.0106660 (PMC4154722; doi:10.1371/journal.pone.0106660)
Supplement: Table S1 — Summary of the features used in this study. (DOC) [file pone.0106660.s011.doc]

**Table S1. Summary of the features used in this study.**

| **Feature types** | **Description** |
| --- | --- |
| Single feature | A vector consisting of pairwise mutation scores between HA1 sequences of virus using single residue information; the mutation score is calculated based on binary or PIMA [1] protein similarity matrix |
| Co-feature | A vector consisting of the inner product of two single features |
| Sinco feature | A combination of single and co features with restrictions on constructing co-features   - sinco+EvolT*m*: also called *co-evolutionary restriction*, only builds co-feature for two residues with normalized mutual information Z score larger than a threshold hold value *m* - sinco+Struct*n*A: also called *co-neighboring restriction*, only builds co-feature for two residues with distance less than n Å in crystal structure of a template HA (pdb file 2VIU) - sinco+EvolT*m+* Struct*n*A: only builds co-feature if the above two restrictions are both satisfied |
