# Supplementary material for: Sequence-Based Antigenic Change Prediction by a Sparse Learning Method Incorporating Co-Evolutionary Information
Source: PLoS One. 2014 Sep 4;9(9):e106660. doi: 10.1371/journal.pone.0106660 (PMC4154722; doi:10.1371/journal.pone.0106660)
Supplement: Table S2 — Compare the influence of restriction method and lasso parameter on average prediction RMSE from 1985 to 2003. Each cell records the average prediction RMSE of the corresponding Lasso parameter, e.g. “2−1” and restriction method, e.g. “Single” on sequential prediction data from 1985 to 2003 (see section “Parameter tuning” in “Materials and Methods” for the definition of sequential prediction). (DOC) [file pone.0106660.s012.doc]

**Table S2. Compare the influence of restriction method and lasso parameter on average prediction RMSE from 1985 to 2003.**

| **Antigenic Drift** | **2^-1^** | **2^0^** | **2^1^** | **2^2^** | **2^3^** | **2^4^** | **2^5^** | **2^6^** | **2^7^** | **2^8^** | **2^9^** |
| --- | --- | --- | --- | --- | --- | --- | --- | --- | --- | --- | --- |
| Single | 1.86 | 1.55 | 1.08 | 0.65 | 0.67 | 0.66 | 0.66 | 0.66 | 0.66 | 0.66 | 0.66 |
| 6A | 1.85 | 1.57 | 1.2 | 0.6 | 0.58 | 0.63 | 0.63 | 0.63 | 0.63 | 0.63 | 0.63 |
| 10A | 1.83 | 1.49 | 1.21 | 0.58 | 0.55 | 0.55 | 0.57 | 0.56 | 0.57 | 0.57 | 0.57 |
| T4 | 1.77 | 1.35 | 1.21 | 0.55 | 0.53 | 0.51 | 0.53 | 0.53 | 0.54 | 0.54 | 0.55 |
| T8 | 1.79 | 1.58 | 1.21 | 0.59 | 0.56 | 0.56 | 0.56 | 0.56 | 0.57 | 0.56 | 0.57 |
| T10 | 1.79 | 1.53 | 1.06 | 0.64 | 0.6 | 0.61 | 0.62 | 0.61 | 0.62 | 0.62 | 0.64 |
| T16 | 1.86 | 1.55 | 1.06 | 0.63 | 0.63 | 0.63 | 0.63 | 0.62 | 0.62 | 0.63 | 0.62 |
| 10A+T2 | 1.83 | 1.49 | 1.21 | 0.61 | 0.58 | 0.55 | 0.56 | 0.55 | 0.55 | 0.55 | 0.55 |

Each cell records the average prediction RMSE of the corresponding Lasso parameter, e.g. “2^-1^” and restriction method, e.g. “Single” on sequential prediction data from 1985 to 2003 (see section “Parameter tuning” in ``Materials and Methods” for the definition of sequential prediction).
