# Supplementary material for: Sequence-Based Antigenic Change Prediction by a Sparse Learning Method Incorporating Co-Evolutionary Information
Source: PLoS One. 2014 Sep 4;9(9):e106660. doi: 10.1371/journal.pone.0106660 (PMC4154722; doi:10.1371/journal.pone.0106660)
Supplement: Table S3 — Compare the influence of restriction methods on antigenic drifts. Each cell records the prediction RMSE of the corresponding restriction method, e.g. “Single” on antigenic drift data, e.g. “HK68-EN72”. To avoid randomness, the RMSE are averaged over 100 runs. In each run, we perform a 5 folder cross validation. For brevity, “6A” indicates co-neighbor restriction with distance 6 angstrom, and “T4” indicates evolutionary restriction with Z-score threshold 4. Similar definition applies for other methods. (DOC) [file pone.0106660.s013.doc]

**Table S3. Compare the influence of restriction methods on antigenic drifts.**

| **Antigenic Drift** | **Single** | **6A** | **10A** | **T4** | **T8** | **T10** | **T16** | **10A+T2** |
| --- | --- | --- | --- | --- | --- | --- | --- | --- |
| HK68-EN72 | 0.72 | 0.70 | 0.70 | 0.56 | 0.55 | 0.56 | 0.57 | 0.70 |
| EN72-VI75 | 0.64 | 0.61 | 0.61 | 0.61 | 0.62 | 0.62 | 0.62 | 0.62 |
| VI75-TX77 | 0.91 | 0.89 | 0.9 | 0.89 | 0.9 | 0.9 | 0.89 | 0.9 |
| TX77-BK79 | 0.55 | 0.5 | 0.51 | 0.49 | 0.51 | 0.51 | 0.52 | 0.52 |
| BK79-SI87 | 0.45 | 0.42 | 0.43 | 0.44 | 0.42 | 0.45 | 0.48 | 0.43 |
| SI87-BE89 | 0.58 | 0.58 | 0.58 | 0.58 | 0.58 | 0.58 | 0.58 | 0.59 |
| BE89-BE92 | 0.88 | 0.86 | 0.86 | 0.86 | 0.87 | 0.88 | 0.87 | 0.86 |
| BE92-WU95 | 0.57 | 0.57 | 0.57 | 0.57 | 0.57 | 0.57 | 0.57 | 0.57 |
| WU95-SY97 | 0.76 | 0.76 | 0.75 | 0.75 | 0.74 | 0.76 | 0.75 | 0.77 |
| SY97-FU02 | 0.94 | 0.93 | 0.93 | 0.93 | 0.93 | 0.93 | 0.94 | 0.93 |
| FU02-CA04 | 1.43 | 1.41 | 1.39 | 1.41 | 1.42 | 1.42 | 1.42 | 1.42 |
| CA04-BR07 | 1.45 | 1.45 | 1.45 | 1.44 | 1.44 | 1.44 | 1.45 | 1.45 |

Each cell records the prediction RMSE of the corresponding restriction method, e.g. “Single” on antigenic drift data, e.g. “HK68-EN72”. To avoid randomness, the RMSE are averaged over 100 runs. In each run, we perform a 5 folder cross validation. For brevity, “6A" indicates co-neighbor restriction with distance 6 angstrom, and “T4" indicates evolutionary restriction with Z-score threshold 4. Similar definition applies for other methods.
