# Supplementary material for: Sequence-Based Antigenic Change Prediction by a Sparse Learning Method Incorporating Co-Evolutionary Information
Source: PLoS One. 2014 Sep 4;9(9):e106660. doi: 10.1371/journal.pone.0106660 (PMC4154722; doi:10.1371/journal.pone.0106660)
Supplement: Table S4 — Compare the influence of lasso parameter on antigenic drifts. Each cell records the prediction RMSE of the corresponding lasso parameter, e.g. “2−1” on antigenic drift data, e.g. “HK68-EN72”. To avoid randomness, the RMSE are averaged over 100 runs. In each run, we perform a 5 folder cross validation. (DOC) [file pone.0106660.s014.doc]

**Table S4. Compare the influence of lasso parameter on antigenic drifts.**

| **Antigenic Drift** | **2^-1^** | **2^0^** | **2^1^** | **2^2^** | **2^3^** | **2^4^** | **2^5^** | **2^6^** | **2^7^** | **2^8^** | **2^9^** |
| --- | --- | --- | --- | --- | --- | --- | --- | --- | --- | --- | --- |
| HK68-EN72 | 0.74 | 0.68 | 0.62 | 0.55 | 0.58 | 0.58 | 0.55 | 0.6 | 0.59 | 0.57 | 0.57 |
| EN72-VI75 | 0.63 | 0.61 | 0.63 | 0.63 | 0.63 | 0.64 | 0.63 | 0.64 | 0.63 | 0.63 | 0.63 |
| VI75-TX77 | 1.04 | 0.89 | 0.92 | 0.91 | 0.91 | 0.9 | 0.91 | 0.91 | 0.9 | 0.91 | 0.91 |
| TX77-BK79 | 0.53 | 0.49 | 0.5 | 0.59 | 0.62 | 0.62 | 0.62 | 0.62 | 0.62 | 0.62 | 0.62 |
| BK79-SI87 | 0.67 | 0.45 | 0.42 | 0.42 | 0.42 | 0.45 | 0.45 | 0.45 | 0.46 | 0.46 | 0.46 |
| SI87-BE89 | 0.66 | 0.58 | 0.59 | 0.59 | 0.59 | 0.59 | 0.59 | 0.58 | 0.58 | 0.58 | 0.58 |
| BE89-BE92 | 1.19 | 0.96 | 0.87 | 0.87 | 0.86 | 0.86 | 0.86 | 0.86 | 0.86 | 0.86 | 0.86 |
| BE92-WU95 | 0.59 | 0.57 | 0.57 | 0.57 | 0.57 | 0.57 | 0.57 | 0.57 | 0.57 | 0.57 | 0.57 |
| WU95-SY97 | 0.92 | 0.78 | 0.74 | 0.76 | 0.79 | 0.81 | 0.8 | 0.78 | 0.79 | 0.78 | 0.78 |
| SY97-FU02 | 1.03 | 0.94 | 0.93 | 0.93 | 0.93 | 0.94 | 0.94 | 0.93 | 0.94 | 0.94 | 0.93 |
| FU02-CA04 | 1.45 | 1.43 | 1.41 | 1.39 | 1.39 | 1.39 | 1.39 | 1.39 | 1.39 | 1.39 | 1.39 |
| CA04-BR07 | 1.45 | 1.44 | 1.44 | 1.44 | 1.44 | 1.44 | 1.44 | 1.44 | 1.44 | 1.44 | 1.44 |

Each cell records the prediction RMSE of the corresponding lasso parameter, e.g. “2^-1^” on antigenic drift data, e.g. “HK68-EN72”. To avoid randomness, the RMSE are averaged over 100 runs. In each run, we perform a 5 folder cross validation.
