# Supplementary material for: Sequence-Based Antigenic Change Prediction by a Sparse Learning Method Incorporating Co-Evolutionary Information
Source: PLoS One. 2014 Sep 4;9(9):e106660. doi: 10.1371/journal.pone.0106660 (PMC4154722; doi:10.1371/journal.pone.0106660)
Supplement: Table S5 — Comparison of 2 machine learning methods Lasso and Ridge regression and 2 scoring schemes 0–1 and PIMA. Each cell lists the smallest average prediction RMSE for all feature types and model parameters on drift data “HK68-EN72” and “BE92-WU95”, and sequential data [1968, 1985], [1968, 1986] and [1968, 1987]. (DOC) [file pone.0106660.s015.doc]

**Table S5. Comparison of 2 machine learning methods Lasso and Ridge regression and 2 scoring schemes 0-1 and PIMA.**

| **Method** | **1968-1985** | **1968-1986** | **1968-1987** | **HK68-EN72** | **BE92-WU95** |
| --- | --- | --- | --- | --- | --- |
| Ridge 0-1 | 0.41 | 0.33 | 0.38 | 0.55 | 0.56 |
| Ridge PIMA | 0.43 | 0.36 | 0.35 | 0.56 | 0.56 |
| Lasso 0-1 | 0.33 | 0.29 | 0.37 | 0.55 | 0.56 |
| Lasso PIMA | 0.33 | 0.28 | 0.25 | 0.54 | 0.56 |

Each cell lists the smallest average prediction RMSE for all feature types and model parameters on drift data “HK68-EN72" and “BE92-WU95", and sequential data [1968, 1985], [1968, 1986] and [1968, 1987].
